# Some Properties of Vulcanite Rubber

**Published:** 1899-01

**Authors:** S. E. Davenport


ARTICLE VIII.

### SOME PROPERTIES OF VULCANITE RUBBER.

DR. S. E. DAVENPORT.

I take the liberty this evening of calling attention to some properties of vulcanite rubber which I think are not generally understood.

These characteristics are principally useful when changes are being made in old places, the additions of new rubber or teeth, or the repairing of fractured plates.

I remember well, when practicing in the country about twenty years ago, a man coming into the office with bottles of some fluid, the application of which to a rubber plate, he said, would enable one to stick new rubber to the old without subsequent cleavage.

My father, in whose office I was, expressed considerable amusement over this, for he had been adding new rubber to old for many years without the use of chemicals.

Unvulcanized rubber, when forcibly pressed with a hot instrument against a clean, dry surface of rubber already vulcanized, will form, when vulcanized, a perfect union with the old rubber, and will break as quickly at any point as at the junction of the new and the old.

Should a rubber plate need a new block or tooth, the old block or tooth having been removed by the application of heat and the pressure of an instrument, a sharp scraper and engine burs should be used to cut away some of the rubber which held the block in position, particularly the portions in which the pins were embedded.

The new block now being ground and fitted for position, the old rubber is again scraped to insure a clean, dry surface, and small pieces of rubber should be spread upon this surface with a flattened instrument repeatedly heated in a flame. An instrument which is not sufficiently hot sticks to the fresh rubber and causes it to drag. When sufficient rubber is stuck to the surface, the block should be heated and pressed to its place, a cloth protecting the hand. Any rubber which may squeeze out should be trimmed off, the plate invested and vulcanized.

Fractured plates should be brought into proper position being held with softened wax on the lingual surface until plaster can be poured upon the surface which fits against the gum.

The thickness of rubber should then be reduced by the use of sharp scrapers and burs, and one or more grooves may be made wherever the plate needs strengthening, for the introduction of pieces of roughened German silver wire.

When the wire shall have been bent and prepared for the grooves referred to, narrow strips of rubber are packed into the grooves with a hot instrument, and the appropriate wire for each groove is warmed and pressed into the new rubber in the groove, the entire surface then being covered, wires and all, with rubber, piece by piece, to whatever thickness necessary, with the hot instrument.

This use of German silver wire in repairing fractured plates is particularly valuable in that it gives the required strength without the necessity of making the plate thick with rubber. Particularly is its use advisable in full dentures if the band be broken above the incisor teeth.

There is almost no limit to the use which may be made of this propriety of rubber, for it can be stuck to a plaster model with a hot instrument almost as well as to a basis of old rubber.

A few weeks ago a lady consulted me concerning a full upper rubber denture, the front teeth of which were too short, and as the plate had been worn a number of years, the suction was imperfect. There were reasons why it did not seem advisable to make a new plate, and as the lady had an older rubber plate which had been discarded some years before, I suggested that she allow me to experiment with that before doing anything to the one she was then wearing.

I first made a small groove all around the palatine surface near the edge of the plate, and packed into the groove with a hot instrument fresh rubber to form a ridge, or "Folsom," another one being made also around the air-chamber.

The surface of the plate was then invested in plaster and the six front teeth removed and replaced, two at a time, so as to be able to judge of their proper length and contour, the sockets being prepared for new rubber, which was packed in and the teeth lengthened as desired. The plate was then invested and vulcanized.

Although this plate had not been worn for at least five years, the suction was perfectly satisfactory fifteen minutes after it was put in the mouth, and while the "Folsom" ridge made the mouth sore and had to be partially scraped away at points two or three different times, within ten days the plate was as comfortable and as serviceable as a new one would have been,

This result being so satisfactory, the other plate was taken in hand and treated in the same manner except that the "Folsom" ridge was not made as high, and the lady did not return after the plate was put in the mouth, she sending me a note instead, assuring me that the plate was comfortable and a success in every way.—*International.*

---

